# Nanostructured Samarium Doped Fluorapatites and Their Catalytic Activity towards Synthesis of 1,2,4-Triazoles

**DOI:** 10.3390/molecules21101281

**Published:** 2016-09-24

**Authors:** Kranthi Kumar Gangu, Suresh Maddila, Surya Narayana Maddila, Sreekantha B. Jonnalagadda

**Affiliations:** School of Chemistry & Physics, University of KwaZulu-Natal, Westville Campus, Chiltern Hills, Durban-4000, South Africa; kkgangu@ymail.com (K.K.G.); sureshmskt@gmail.com (S.M.); maddilasurya@gmail.com (S.N.M.)

**Keywords:** Sm-fluorapatite, nanostructured catalysts, 1,2,4-traizole, organic modifiers, textural properties, amino acids

## Abstract

An investigation was conducted into the influence of the amino acids as organic modifiers in the facile synthesis of metal incorporated fluorapatites (FAp) and their properties. The nanostructured Sm doped fluorapatites (Sm-FAp) were prepared by a co-precipitation method using four different amino acids, namely glutamic acid, aspartic acid, glycine and histidine. The materials were characterized by various techniques including X-ray diffraction (XRD), Fourier transform infra-red spectroscopy (FT-IR), field emission scanning electron microscopy (FE-SEM), energy-dispersive X-ray spectroscopy (EDX), high resolution transmission electron microscopy (HR-TEM), N_2_-adsorption/desorption isotherm, temperature programmed desorption (TPD) and fluorescence spectrophotometry. Under similar conditions, Sm-FAp prepared using different amino acids exhibited distinctly different morphological structures, surface area and pore properties. Their activity as catalysts was assessed and Sm-FAp/Glycine displayed excellent efficiency in the synthesis of 1,2,4-triazole catalyzing the reaction between 2-nitrobenzaldehyde and thiosemicarbazide with exceptional selectivity and 98% yield in a short time interval (10 min). The study provides an insight into the role of organic modifiers as controllers of nucleation, growth and aggregation which significantly influence the nature and activity of the catalytic sites on Sm-FAp. Sm-FAp could also have potential as photoactive material.

## 1. Introduction

In recent years, due to their exceptional properties the application of calcium apatites, Ca_10_(PO_4_)_6_(OH)_2_ (HAp), particularly fluorapatites, Ca_10_(PO_4_)_6_F_2_ (FAp) has become far-reaching in the fields of biomedicine, bioactive implant surfaces, dental implants, catalysis, drug delivery and environment engineering [1,2,3,4,5,6]. FAp crystallizes in a hexagonal crystal system with space group *P6*/*m* (175) having lattice parameters *a* = 9.3684 Å and *c* = 6.8841 Å in which the calcium and phosphate ions are arranged around the fluoride ions in the [001] direction (along c-axis) [7,8]. Tuning the composition and surface properties of FAp imparts flexibility with respect to behavior as catalysts or as supports for several catalytic centers which accelerate many value-added organic transformations [9,10,11]. Studies have demonstrated that apatites can be utilized in the development of sustainable, green and reliable heterogeneous catalysts in the organic transformations such as Michael addition, Knoevenagel condensation, oxidation, hydroformylation, cyclic additions and epoxidation reactions [12,13,14,15,16]. The ability of FAp to adsorb organic, inorganic and metal salt substrates on their surfaces make them ideal for several catalytic applications. Due to the presence of both Brønsted/Lewis acidic and basic sites on its surface, FAp provides multiple active sites for catalysis [17]. The acidic sites are generated from the Ca^2+^ and phosphorous of PO_4_ group, whereas the basic sites are obtained from the F^−^ and oxygen of PO_4_ group. The electron rich Lewis basic sites (P-O^−^ and F^−^) and acidic sites (M^2+^, PO_2_H^2+^) which are closely packed in different locations in the FAp surfaces can facilitate the organic transformations through the acid-base catalyzed processes [18,19,20]. Gruselle et al. in their study have reported that the hydroxyl groups present on the surfaces of calcium hydroxyapatite played a key role in the Michael C-C bond formation [21]. In order to improve FAp as a heterogeneous catalyst, it can be modified by the partial replacement of Ca^2+^ with other divalent or trivalent ions, which lead to the creation of well-defined Lewis acidic active sites on the surfaces. Rare earth elements from La to Lu possess ionic radii comparable to Ca^2+^ making such replacement much easier [22,23,24]. In addition, the incorporation of rare earth elements, particularly samarium, into the FAp structure increases wide ranging catalytic properties [25,26,27,28]. The catalytic efficiency of FAp also relies on its physical features. Thus, synthesis of such well-crafted materials with specific size, controlled morphology and hierarchical architecture is of real significance [29,30,31]. The synthesis of such materials can be influenced by various factors including the nature of, surfactants, capping agents, chelating agents or organic compounds employed [32,33]. Polymers-assisted synthesis can lead to varied morphologies, restriction of nucleation and confiscation of crystal growth [34,35]. Amino acids as crystal growth modifiers are an attractive option in the preparation of modified fluorapatites, in terms of green principles and biocompatibility as they are eco-friendly, sustainable and cost-effective. They possess functional groups (–NH_2_ & –COOH) with capability to tune the size and dimensions of optimistic materials, while their side chains (-R group) remain intact [36,37,38,39]. The literature has few reports on the scope and role of crystal growth modifiers in the synthesis of fluorapatites [40]. 

The efficacy of the nanostructured Sm loaded FAp as catalysts for the synthesis of 1,2,4-triazoles was assessed. Triazoles have plentiful applications in the biological and pharmaceutical fields [41,42,43]. Appropriate intrusion of the 1,2,4-triazole nucleus in biologically active compounds is known to create anti-tubercular, anti-malarial, anti-microbial, anti-inflammatory, anti-tumor and anti-convulsant activities [44,45]. In addition to their pharmaceutical importance, high nitrogen containing compounds like 1,2,4-triazoles are extensively used as energetic materials due to their valuable ballistic properties [46]. The above applications highlight the importance of a facile synthetic route for triazoles to surmount the hindrances such as elaborate reaction conditions, high temperature maintenance, long work-up procedures and low yields. In the earlier studies, we have reported the synthesis and characterization of diverse metal (Fe Co, Pt, Sr, Ir, and V) substituted hydroxyapatites and their scope as heterogeneous catalysts in value-added organic transformations such as selective oxidation of n-pentanes and in Knoevenagel condensation reactions with excellent yields [47,48,49,50]. We have also reported the scope of Sm(III) based coordination polymer as a viable yellow light emitter [51].

In this paper, we report the amino acids-assisted synthesis of nanoparticles of fluorapatites impregnated with Sm through co-precipitation methods with examination of the changes in the physical characteristics such as size, morphology, surface area, pore properties and fluorescence activity of the products by using four different amino acids as crystal growth modifiers. The activities of the materials as catalysts for the reaction between 2-nitro-benzaldehyde and thiosemicarbazide to yield thio-triazole moiety are compared.

## 2. Results and Discussion

### 2.1. XRD Analysis 

**Figure 1:**
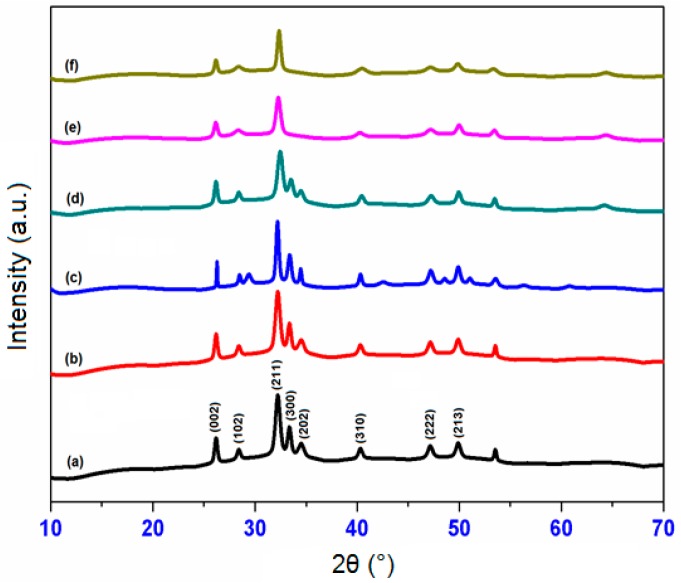
X-ray diffraction pattern of (**a**) FAp/without amino acid; (**b**) Sm-FAp/without amino acid; (**c**) Sm-FAp/Glutamic acid; (**d**) Sm-FAp/Aspartic acid; (**e**) Sm-FAp/Glycine; (**f**) Sm-FAp/Histidine.

To investigate the crystalline phases of Sm-FAp, X-ray diffraction measurements were performed and the results are illustrated in Figure 1. The diffraction patterns of the six samples are well matched with the fluorapatite standard (JCPDS # 15-0876). No significant variation is observed in the diffraction pattern of samples prepared with different amino acids and no characteristic peaks of impurities are observed, which confirms that the present experimental conditions used are suitable for the preparation of Sm-FAp. The peaks (211), (300) and (202) have high intensities with glutamic acid (Figure 1c) and aspartic acid (Figure 1d), but those peaks are missing with the other two amino acids, glycine and histidine. This is ascribed to the variance in the texture due to different amino acids used and preferred orientation of those planes which probably have not allowed appropriate diffraction to occur. No additional phases are found in the patterns indicating that the doped substrate is effectively built into the host lattice. When compared with the FAp sample without amino acid, the peaks of remaining samples appear to be broadened, suggesting a relative decrease in the crystallite size, which is confirmed by the crystallite size calculated by Scherrer’s formula using (211) reflection of each XRD pattern. The average crystallite size of all samples are summarised in Table 1. The amino acid introduction during the synthesis was observed to induce a notable modification in the crystallite size by its surface capping ability. The similar observation was made by Pallazzo et al. when varied amino acids were used in the preparation of hydroxyapatite nanaocrystals, where amino acid altered the morphology of crystals as well as functionalization the hydroxyapatite surface [52]. The degree of crystallinity of the samples in the current study was examined after successive profile fit processes using High-score plus software and the results reveals that when compared with un-calcined XRD patterns, the crystallinity percentage was improved after calcination (Figure S1). Furthermore, the degree of crystallinity for the un-doped FAp (65% ± 2%) and Sm-FAp (63% ± 2%) in absence of amino acid were slightly higher than the amino acid assisted Sm-FAp (55%–60% ± 5%), which indicates that the presence of amorphous amino acid decrease the crystallinity in amino acid assisted Sm-FAp. 

### 2.2. FT-IR Analysis 

**Figure 2:**
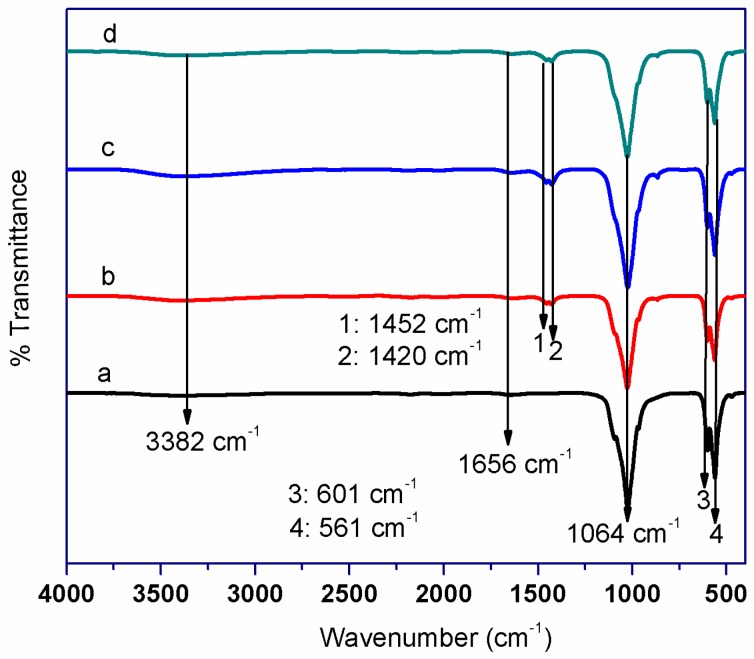
FT-IR spectra of (**a**) Sm-FAp/Glutamic acid; (**b**) Sm-FAp/Aspartic acid; (**c**) Sm-FAp/Glycine (**d**) Sm-FAp/Histidine.

Figure 2 illustrates the FT-IR spectra for the formation of Sm-FAp in the presence of different amino acids. No changes are observed in the spectra of Sm-FAp prepared using different amino acids. This plainly suggests that amino acids contribute to the modification of structural features without major changes in the final product. The amino acids played a role in the crystal growth and surface modification throughout the reaction. The major absorption peaks at 1064 cm*^−^*^1^, 601 cm*^−^*^1^ and 561 cm*^−^*^1^ indicate the phosphate group’s stretching and bending vibrations. The peaks at 601 and 561 cm*^−^*^1^ are from the bending asymmetric modes of triply degenerated O–P–O bonds of phosphate group, whereas the band at 1064 cm*^−^*^1^ is assigned to P–O asymmetric stretching vibrational mode of phosphate group [53]. The observed bands at 1656 and 1452 cm^−1^ respectively correspond to C=O stretching and COO– asymmetric vibrations of carboxylic acid group present in the amino acid. The peaks at 3382 and 1420 cm*^−^*^1^ respectively are related to the N–H and NH_2_–H^+^ symmetric stretching vibrations of amino group. No peaks at 1452 and 1420 cm*^−^*^1^ were observed for the glutamic acid assisted Sm-FAp and the weak peaks appeared at 1656 cm*^−^*^1^ and 3382 cm*^−^*^1^ indicate minimal presence of amino acid on the surface (Figure 2a). The absorption bands reflecting the presence of amino acid suggest that it binds to the Sm-FAp surface effectively. 

### 2.3. SEM and TEM Analysis of Sm-FAp and the Influence of Amino Acids

**Figure 3:**
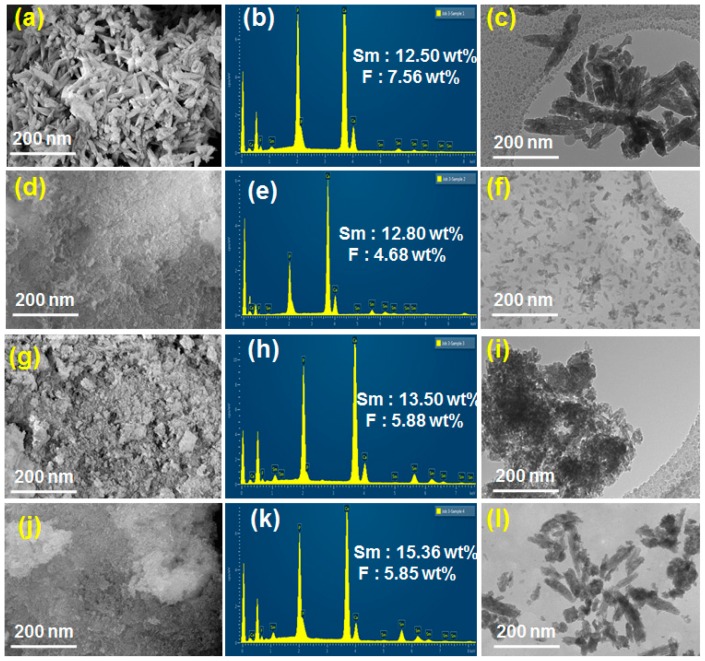
FE-SEM, HR-TEM micrographs and EDX spectra of (**a**–**c**) Sm-FAp/Glutamic acid; (**d**–**f**) Sm-FAp/Aspartic acid; (**g**–**i**) Sm-FAp/Glycine; (**j**–**l**) Sm-FAp/Histidine.

The morphology, size and shape of the particle characteristics were obtained from FE-SEM and HR-TEM micrographs. Figure 3 shows the SEM and TEM micrographs of Sm-FAp with various amino acids. SEM and TEM micrographs reflect the effect of amino acids on change of morphological features and size of the particles. When glutamic acid was used as crystal growth modifier, the crystals have a flower bud-like shape (Figure 3a) attaining dimensions of 30–40 nm in length and 20–25 nm in diameter. The TEM micrograph (Figure 3c) show the particles are in bunches with sharp ended edges. In the case of aspartic acid, unlike glutamic acid, the particles are widely dispersed with smoother surfaces (Figure 3d) and 40–60 nm in length with 20–40 nm diameters as shown in TEM micrograph (Figure 3f). The TEM micrograph also reveals that particles are well dispersed. With glycine also similar features are observed, but the particles had rough and compact morphological surfaces (Figure 3g). A perusal of TEM micrograph indicates that the particles are 14–35 nm in length and 8–10 nm in diameter (Figure 3i). In the presence of histidine, the size distribution of discrete nanoparticles is very similar to that with the aspartic acid and exhibit similar surface morphology (Figure 3j), but particle dimensions are different. The TEM micrograph of Sm-FAp with histidine (Figure 3l) shows particles 100–160 nm in length and 35–50 nm in diameter. Figure 3b,e,h,k shows the EDX spectra of all products, which endorses the existence of doped samarium and also confirms the formation of nanostructures of Sm-FAp together with the presence of Sm, Ca, P and F in the specimen. The existence of fluorine and doping samarium was further confirmed by the ICP-OES analysis and the correlated results with EDX are tabulated in Table S1.

### 2.4. Textural Properties of Sm-FAp with Influence of Amino Acids

**Figure 4:**
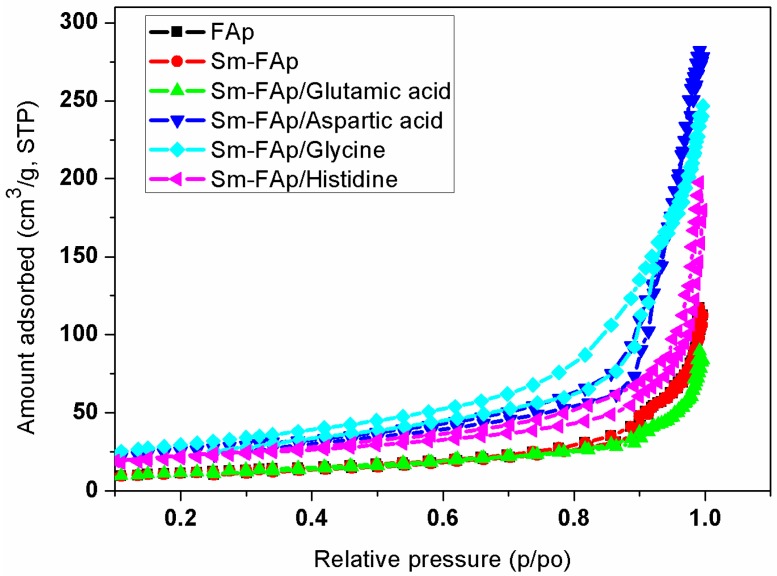
N_2_ adsorption/desorption isotherm at 77 K.

The textural properties pertaining to a series of Sm-FAp with various amino acids were determined using adsorption/desorption isotherms of nitrogen under cryogenic conditions. The prime objective was to investigate the effect of varied amino acids in the synthesis of Sm-FAp on the BET-surface area, pore volume and pore sizes. The isotherms for all the samples match well with Type IV isotherm (Figure 4) and the quantity of nitrogen adsorbed was found to be different for different products. Table 1 summarises the trends in BET-surface area, pore volume and pore sizes in presence of different amino acids. With glycine, the sample shows the highest surface area of 106.96 m^2^/g with decreasing order as follows: aspartic acid > histidine > glutamic acid. Average pore volume was highest with aspartic acid and lowest with glutamic acid-assisted sample. While all products show a meso pore size range of 13 to 18 nm and aspartic acid-assisted sample displayed highest pore size. The lower surface area recorded with un-doped FAp and Sm-FAp in absence of amino acid confirms the importance of amino acid on their surface modifications. The textural properties of samples are related to the physical parameters such as morphology, particle size etc. The lowest particle size of Sm-FAp/Glycine possessed the highest surface area, similar to that of Sm-FAp/Aspartic acid. Although Sm-FAp/Glutamic acid contained smaller particles to Sm-FAp/Histidine, the later displayed higher surface area. This may be attributed to the wide distribution of particles with morphological structure and surfacial features of Sm-FAp/Histidine similar to Sm-FAp/Aspartic acid. 

### 2.5. Fluorescence Activity of Sm-FAp with Amino Acids

**Figure 5:**
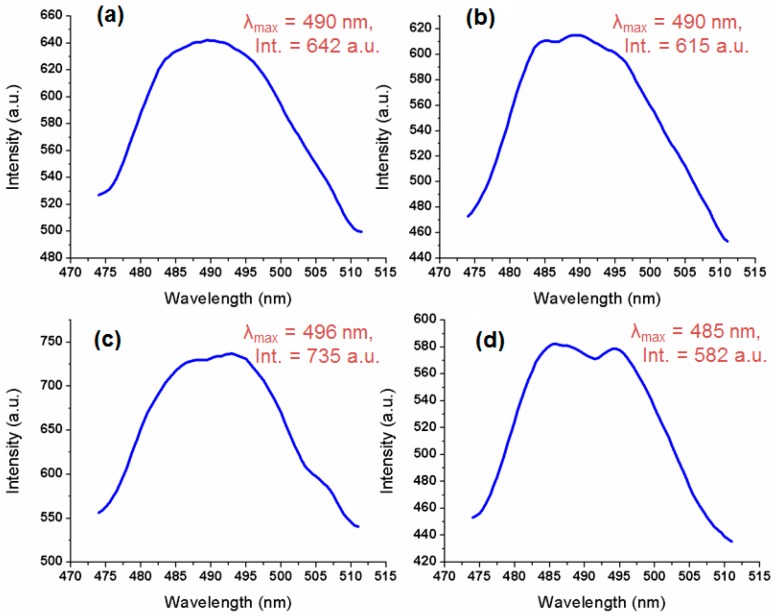
Fluorescence spectra of (**a**) Sm-FAp/Glutamic acid; (**b**) Sm-FAp/Aspartic acid; (**c**) Sm-FAp/Glycine; (**d**) Sm-FAp/Histidine.

**Figure 6:**
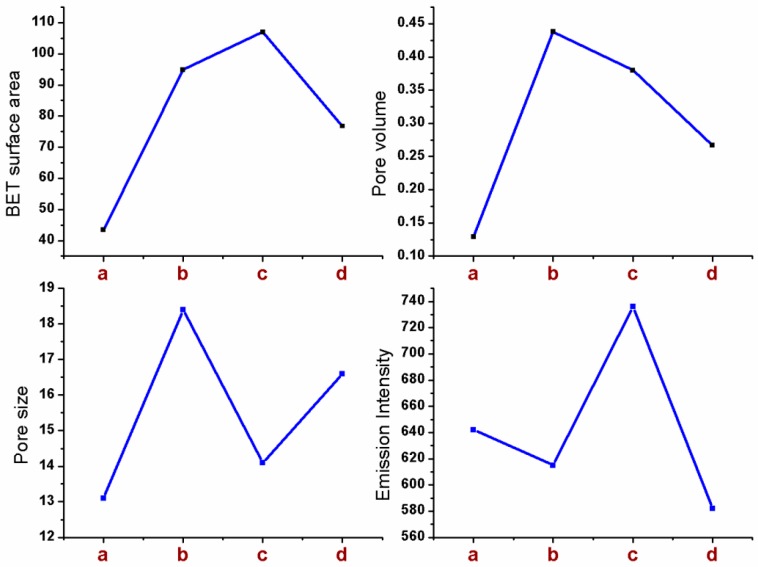
A perspective view of effect of amino acids on BET surface area, pore volume, pore size and emission intensity ((**a**) Sm-FAp/Glutamic acid; (**b**) Sm-FAp/Aspartic acid; (**c**) Sm-FAp/Glycine; (**d**) Sm-FAp/Histidine).

Lanthanides are well-known fluorescents, but their fluorescence efficacy purely depends on the selection of plausible host matrix [54]. Fluorapatites are promising supporting materials and possess capability to host the varied lanthanides. The presence of Ca^2+^, F and PO_4_^3*−*^ ions in FAp could not initiate any luminescence, since no defect/impurity-related color centers were stabilized in its lattice. FAp doped with rare earth (RE) or transition metal ions have the ability to cause luminescence due to electronic transitions existing between the doping metal orbital [55]. Samarium impregnation into FAp showed a fairly wide and strong emission bands in visible region upon excitation at 254 nm and it was attributed to 4f-orbital transitions of samarium entity [56,57,58]. In this study, emission peaks of amino acid-assisted synthesis of Sm-doped FAp nanostructures have predominantly showed a change in the intensity with the amino acid used (Figure 5). The maximum emission wavelength (λ_max_) of Sm-FAp in the presence of glutamic acid and aspartic acid was assigned to 490 nm with intensity of 642 and 615 units, respectively. In the case of glycine, λ_max_ shifted slightly towards the higher wavelength side at 496 nm with high emission intensity of >735 units. On the other hand, histidine-assisted sample exhibited wavelength maximum at shorter wavelength 485 nm, but with lower intensity with 582 units. Ultimately, the results demonstrate that the change in size, shape and textural traits occur due to amino acids assisted synthesis also influence fluorescence activity. This could lead to new applications through modulating the physical characteristics of samples. Figure 6 illustrates the impact of amino acids as crystal growth modifiers in the synthesis of Sm-FAp. The change in emission intensities with respect to amino acid used has direct relationship to the particle size attained by the Sm-FAp. The emission intensity is inversely proportional to the particle size of Sm-FAp. The highest emission intensity from samples of Sm-FAp/Glycine, which possess lower particle size relative to other amino acid assisted Sm-FAp samples. The luminescence activity was found to be profoundly sensitive to particle size and can be intensified by the reduction of particle size by tailoring the interfaces with the use of organic modifiers [59,60]. The particle size of Sm-FAp was found to decrease in the following order of amino acid used: Sm-FAp/Histidine > Sm-FAp/Aspartic acid > Sm-FAp/Glutamic acid > Sm-FAp/Glycine, while the order of emission intensity was Sm-FAp/Histidine < Sm-FAp/Aspartic acid < Sm-FAp/Glutamic acid < Sm-FAp/Glycine.

### 2.6. Catalytic Activity

The effect of the four catalysts on the reaction of 2-nitro benzaldehyde with thiosemicarbazide to yield 1,2,4-triazole moiety was investigated. In a typical reaction, equivalent molar proportions of 2-nitro benzaldehyde (1.0 mmol) and thiosemicarbazide (1.0 mmol) were dissolved in 5.0 mL ethanol at room temperature, with continuous stirring. An appropriately weighed (20 mg) of the selected amino acid modified Sm-FAp was added as catalyst to the reaction mixture. The reaction was monitored with TLC at regular time intervals. After confirming the completion of the reaction, the solid catalyst was separated cautiously from the organic liquid product by filtration. The same procedure was repeated for all the other catalysts. Among the catalysts, Sm-FAp/Glycine proved superior. While the uncatalyzed reaction under similar reaction conditions took 2 h with 60% yield, the Sm-FAp/Glycine reaction took 10 min with 98% yield. All the four reactions gave same product, but other three catalysts gave relatively lower yields and had longer reaction times. In order to confirm the need for Sm doping in the catalyst preparation, the reaction was carried out with un-doped FAp in the absence of amino acid. When un-doped FAp was used as catalyst, the reaction took 35 min with 80% yield (Table 2). Sm-FAp/Glycine with a high specific surface area and low particle size proved a better catalyst compared to the other three amino acid assisted Sm-FAp catalysts. The final products were characterized by IR, ^1^H-NMR, ^15^N-NMR and ^13^C-NMR for confirmation of their structures. In the FT-IR spectrum (Figure S2) of final product shows absorption frequencies at 3443, 3321, 3175 cm*^−^*^1^ which indicate the stretching vibrations of three –NH groups present in the 1,2,4-triazole moiety. In addition to this, a stretching vibrational frequency at 1276 cm*^−^*^1^ confirms the presence of bonding between carbon and sulphur (–C=S). Further, the observed signals of ^1^H-NMR at δ 8.10, 8.37, 11.72 ppm indicate the presence of three –NH secondary amine protons (Figure S3), which was confirmed by ^15^N spectrum (Figure S4). In ^13^C-NMR, the signal at δ 178 ppm indicates the thiocarbonyl entity in the structure (Figure S5). 

**Figure 7:**
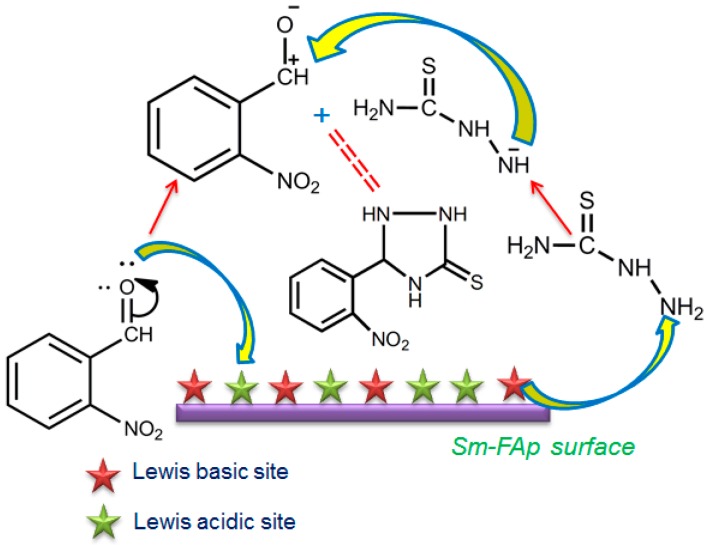
Perspective view of Sm-FAp surfaces facilitating the reaction.

The reaction probably occurs on the surface of Sm-FAp rather than inside cages of the catalyst. The surface provides Lewis acidic and basic sites to catalyze the reaction. The C-N bond can be formed by the nucleophilic addition of thiosemicarbazide to a carbonyl group of 2-nitrobenaldehyde. The Lewis or Brønsted basic sites on the surface of Sm-FAp facilitate the formation of nucleophilic center in the thiosemicarbazide. The reaction is followed by the attack of the nucleophilic center by the electrophile in the carbonyl group in order to form a bond (Figure 7). The doping of samarium on FAp enriches the Lewis acidic centers on surfaces of Sm-FAp, which generate more active sites for the formation of electrophilic center in the carbonyl group. The high specific surface area containing Sm-FAp/Glycine enhances the number of catalytic active sites which make it a better catalyst. The TPD results indicate the existence of acidic sites on the surfaces of the catalysts, Sm-FAp/Glycine being the most acidic (Table 1), in accordance with the catalytic performance exhibited by Sm-FAp/Glycine relative to the other catalysts. The catalytic performances were statistically tested with ANOVA and results are statistically significant (Table S2).

**Scheme 1:**
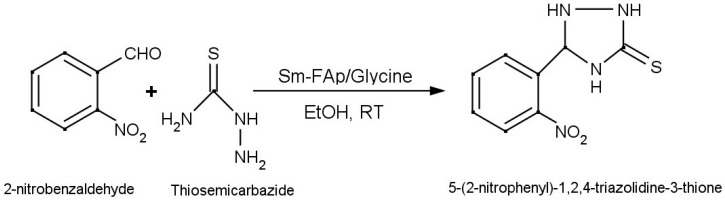
Typical reaction.

Rathinam et al. have reported the preparation of 5-aryl-1,2,4-triazolidine-3-thione and its derivatives using polyethylene glycol as solvent [61]. Jayavant et al. have prepared the 1,2,4-triazolidine-3-thiones using substituted thiosemicarbazide and aldehydes with ionic liquid, [C_16_MPy]AlCl_3_Br [62] as medium. Mane et al. have prepared 5-aryl-1,2,4-triazolidine-3-thiones in the presence of sulfamic acid as catalyst from the mixture of aldehyde, hydrazine hydrate, and trimethylsilyl isothiocyanate [63]. Survey of the literature suggests that 5-(2-nitrophenyl)-1,2,4-triazolidine-3-thione synthesized in this study is a new compound. Furthermore, there were no reports of 1,2,4-triazoles or in any other organic syntheses using Sm-FAp as catalysts (Scheme 1). To evaluate the optimum quantity of Sm-FAp/Glycine required, the reaction was carried out with varied amounts of catalyst from 10 mg to 50 mg. Results show that 20 mg of catalyst is optimal to achieve maximum benefit under the given conditions (Table S3). To assess the efficacy of the process a series of reactions were conducted with different substituted aromatic aldehydes as precursors under the optimized conditions. Sm-FAp/Glycine proved an excellent catalyst in all reactions and produced respective thio-1,2,4-triazoles with impressive yields (Table 3, entries 1–4).

After the first cycle, the catalyst material was recovered thorough vacuum filtration and washed with water, followed by drying in vacuum for 2 h at 60 °C. The activity of recovered catalyst was tested for further cycles. The catalyst displayed excellent efficiency for five cycles with <2% loss of activity. Other amino acid assisted Sm-FAp showed about 6%–9% decrease in activity by the 5th cycle. The recovery and reusability of the catalyst with good catalytic performance is an added advantage in synthesis. 

## 3. Materials and Methods 

### 3.1. Materials

All analytical grade chemicals were purchased and used without any further purification. Calcium nitrate, Ca(NO_3_)_3_·4H_2_O was purchased from Merck (Darmstadt, Germany), Samarium nitrate, Sm(NO_3_)_3_·6H_2_O was procured from Sigma-Aldrich (St. Louis, MO, USA), Amino acids (glutamic acid, aspartic acid, glycine, histidine) were purchased from BDH chemicals Ltd (Poole, UK). Other reactants such as tri-sodium phosphate Na_3_(PO_4_)·12H_2_O and sodium fluoride, NaF were purchased from Merck. Water used in all experiments is highly purified deionized water. 

### 3.2. Preparation of Sm-FAp by Using Amino Acids

A typical solution of 25.0 mL deionized water containing the mixture of 1.5 mmol Na_3_PO_4_·12H_2_O (0.570 g), 2.0 mmol of glutamic acid (0.330 g) and 0.5 mmol of NaF (0.020 g) were stirred continuously until a clear solution was obtained. After adjusting the pH of the solution to 11–11.5 with 1 M NaOH, 2.5 mmol of Ca(NO_3_)_3_·4H_2_O (0.5903 g) was added with vigorous stirring. After few minutes, 5.0% molar concentration of Sm(NO_3_)_3_·6H_2_O (0.074 g) was added slowly to the solution followed with continuous stirring for about 5 h. The beaker was then allowed to stand for 10 min and then centrifuged at 2000 rpm followed by several washings with deionized water. After decanting the supernatant solution, the samples were collected and dried. The dried samples were calcinated to 350 °C for 3 h in a flow of air. The same procedure was repeated for the preparation of other three samples using either 2.0 mmol of aspartic acid (0.266 g), glycine (0.150 g) or histidine (0.310 g). All the four synthesized materials were characterized. Table 4 summarizes the details of the sample proportions. 

### 3.3. Characterization

The synthesized products were investigated by powder X-ray diffractometry (Bruker D8 Advance diffractometer with Ni filtered Cu Kα radiation, V = 45 kV, I = 40 mA) for identifying the phase and crystallinity. The spectra were recorded on a preferential scan rate of 2°/min and a step size of 0.02° in a 2θ range of 10° to 70° at room temperature. FT-IR was conducted on Perkin Elmer, Spectrum 100 spectrometer (Waltham, MA, USA) over a range of 4000 to 400 cm^−1^ at resolution of 4 cm^−1^ to detect the phosphate bands for confirmation of the formation of fluorapatites. Morphology, size and nanostructure of the products were investigated using a field emission scanning electron microscope (FESEM, Zeiss Ultra Plus, Göttingen, Germany) operating at 5–20 kV and a high resolution transmission electron microscope (HRTEM, JEOL 2100, Hitachi, Japan) with an accelerating voltage of 200 kV. All samples were coated with an Au film to get a good resolution for FESEM. The identification and confirmation of fluorine and samarium in the products was carried out by the elemental analysis through Electron dispersive X-ray (EDX, AZtec software, Oxford instruments, Oxon, UK) spectrometer unit, attached to FESEM. The Sm loading was further determined by using Perkin Elmer Inductively coupled plasma Optical Emission Spectrometer (ICP-OES, Shelton, CT, USA) Optima 5300 DV. Prior to ICP analysis, samples were solubilized in aqua regia and homogenized by a microwave digestion process. Textural properties, surface area and pore properties of products were determined by N_2_ sorption isotherm using Micromeritics TriStar II 3020, Norcross, GA, USA at 77 K. Before sorption measurement, all samples were promptly degassed whole night at 180 °C to eliminate the space occupied by unwanted gases. The TPD experiments were conducted on Micromeritics Autochem 2020, Norcross, GA, USA to determine the acidic sites on the surface of the catalysts. Before the conduct of TPD analysis, the samples were pretreated at 200 °C under the helium flow to evacuate the pores. Fluorescence measurements were performed using Perkin Elmer, LS-55 fluorescence spectrophotometer (Waltham, MA, USA). Spectral data such as ^1^H-NMR and ^13^C-NMR for synthesized organic products were recorded using a Bruker Advance 400 spectrometer (Salt Lake city, UT, USA) at ambient temperature. 

## 4. Conclusions 

In summary, nanostructured Sm doped fluorapatites were prepared by a co-precipitation method employing four different amino acids as growth modifiers. Amino acid assisted synthesis showed varied characteristics in term of morphology, size, surface area and pore properties of the Sm-FAp. The glycine-assisted sample exhibited the highest surface area relative to the other amino acids-assisted samples. All samples exhibited desirable fluorescence, but their maximum emission wavelength intensity varied with amino acid being used. Of all samples, the glycine-assisted sample displayed the highest emission intensity at longer wavelengths, while histidine assisted sample showed a blue shift, but with lower intensity. Amino acids used for growth modifications in the synthesis of materials promptly alter the physical characteristics and which finally affect the properties as well as the possible application of the materials. Glycine-assisted Sm-FAp showed the best catalytic activity towards value added organic transformations of aromatic aldehyde to 1,2,4-triazole moiety with 98% yield. Structural features of the catalysts play a prominent role and the choice of application of those materials greatly depends upon their physical characteristics. 

## Figures and Tables

**Table 1:** BET-Surface area, pore properties and TPD data of catalysts.

Sample	BET-Surface Area (m^2^/g)	Pore Volume (cm^3^/g)	Pore Size (nm)	Average Crystallite Size from XRD (nm)	Acidity (mmol NH_3_/g)
FAp/without amino acid	32 ± 1.3	0.13 ± 0.02	11.5 ± 0.2	17.52 ± 0.4	205 ± 6.7
Sm-FAp/without amino acid	39 ± 3.1	0.21 ± 0.02	12.6 ± 0.3	15.58 ± 0.4	271 ± 6.2
Sm-FAp/Glutamic acid	43 ± 1.1	0.22 ± 0.02	13.1 ± 0.2	9.8 ± 0.3	349 ± 12.6
Sm-FAp/Aspartic acid	94 ± 1.5	0.44 ± 0.03	18.4 ± 0.3	6.2 ± 0.4	412 ± 11.4
Sm-FAp/Glycine	106 ± 1.3	0.38 ± 0.01	14.1 ± 0.3	5.7 ± 0.4	607 ± 14.4
Sm-FAp/Histidine	76 ± 1.3	0.27 ± 0.01	16.6 ± 0.3	10.6 ± 0.4	375 ± 11.5

**Table 2:** Catalytic performance of catalysts in the typical reaction ^a^.

Catalyst	Time (min)	Yield (%)
Without catalyst	120	60
Un-doped FAp (without amino acid)	35	80
Sm-FAp/Glutamic acid	15	94
Sm-FAp/Aspartic acid	10	95
Sm-FAp/Glycine	10	98
Sm-FAp/Histidine	20	93

^a^ Reaction conditions: 2-nitro benzaldehyde (1.0 mmol), thiosemicarbazide (1.0 mmol), catalyst (20 mg) and ethanol (5.0 mL) were stirred at room temperature.

**Table 3:** List of synthesis of 1,2,4-triazolidine-3-thione moiety with different substituted aromatic aldehydes with Sm-FAp/Glycine as heterogeneous catalyst *.

Entry	Aldehyde	Product	Yield %	Time (min)
1	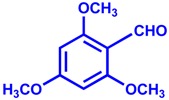	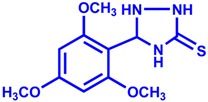	92	15
2	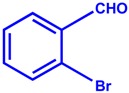	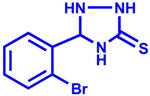	93	20
3	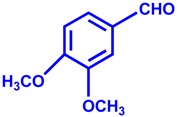	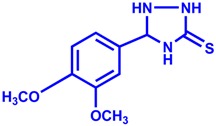	96	15
4	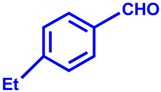	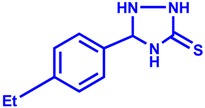	94	10

* Reaction conditions: substituted benzaldehyde (1.0 mmol), thiosemicarbazide (1.0 mmol) catalyst and ethanol (5.0 mL) were stirred at room temperature.

**Table 4:** Chemical composition of amino assisted Sm-FAp.

Sample	Ca(NO_3_)_3_·4H_2_O (mmol)	Sm(NO_3_)_3_·6H_2_O	Na_3_PO_4_·12 H_2_O (mmol)	NaF (mmol)	Amino Acid (2.0 mmol)
**a**	2.5	0.074 g	1.5	0.5	Glutamic acid (0.330 g)
**b**	2.5	0.074 g	1.5	0.5	Aspartic acid (0.266 g)
**c**	2.5	0.074 g	1.5	0.5	Glycine (0.150 g)
**d**	2.5	0.074 g	1.5	0.5	Histidine (0.310 g)

## Supplementary Materials

The following are available online at www.mdpi.com/1420-3049/21/10/1281/s1, Table S1: Elemental analysis by EDX and ICP-OES, Table S2: Statistical report using ANOVA, Table S3: Optimization of the amount of Sm-FAp/Glycine as catalyst in the model reaction^a^, Figure S1: X-ray diffraction pattern of (**a**) FAp/without amino acid; (**b**) Sm-FAp/without amino acid; (**c**) Sm-FAp/Glutamic acid; (**d**) Sm-FAp/Aspartic acid; (**e**) Sm-FAp/Glycine; (**f**) Sm-FAp/Histidine before annealing at 350 °C, Figures S2–S5: FT-IR, ^1^H-, ^15^N-, ^13^C-NMR spectra of 5-(2-nitrophenyl)-1,2,4-triazolidine-3-thione, Figures S6–S17: ^1^H-, ^13^C-, ^15^N-NMR spectra of entries 1–4.
