# Supplementary material for: Nanostructured Samarium Doped Fluorapatites and Their Catalytic Activity towards Synthesis of 1,2,4-Triazoles
Source: Molecules. 2016 Sep 24;21(10):1281. doi: 10.3390/molecules21101281 (PMC6273585; doi:10.3390/molecules21101281)
Supplement: Supplementary file 1 [file molecules-21-01281-s001.pdf]

Supplementary Materials: Nanostructured Samarium Doped Fluorapatites and Their Catalytic Activity towards Synthesis of 1,2,4-Triazoles

Kranthi Kumar Gangu, Suresh Maddila, Surya Narayana Maddila and Sreekantha B. Jonnalagadda

Table S1. Elemental analysis by EDX and ICP-OES

Catalyst	EDX (wt %)		ICP-OES (wt %)	
	F	Sm	F	Sm
Sm-FAp/Glutamic acid	7.56 ± 1.1	12.50 ± 0.8	6.85 ± 0.3	12.37 ± 0.7
Sm-FAp/Aspartic acid	4.68 ± 0.6	12.80 ± 0.3	4.87 ± 0.3	12.59 ± 0.4
Sm-FAp/Glycine	5.88 ± 0.3	13.50 ± 0.4	5.75 ± 0.6	12.95 ± 0.4
Sm-FAp/Histidine	5.85 ± 0.5	15.36 ± 1.2	5.79 ± 0.2	14.90 ± 0.6

Table S2. Statistical report using ANOVA (Single Factor).

**Summary**						
**Groups**	**Count**	**Sum**	**Average**	**Variance**		
Reaction time (min)	6	210	35	1820		
Yield (%)	6	520	86.66667	209.4667		
**ANOVA**						
**Source of Variation**	**SS**	**df**	**MS**	**F**	**P-value**	**F crit**
Between Groups	8008.333	1	8008.333	7.892057	0.018498	4.964603
Within Groups	10147.33	10	1014.733			
Total	18155.67	11				

Table S3. Optimization of the amount of Sm-FAp/Glycine as catalyst in the model reaction [a].

Entry	Catalyst (mg)	Time (min)	Yield (%)
1	10	20	80
2	20	10	98
3	30	10	95
4	40	15	93
5	50	15	91

[a] Reaction conditions: 2-nitro benzaldehyde (1.0 mmol), thiosemicarbazide (1.0 mmol) Sm-FAp/Glycine (20 mg) and ethanol (5.0 mL) were stirred at room temperature.

Products Characterization Data

Scheme 1: *5-(2-Nitrophenyl)-1,2,4-triazolidine-3-thione*: ^{1}H-NMR (400 MHz, DMSO-d_6) δ 7.61 (t, *J* = 8.12 Hz, 1H, ArH), 7.72 (t, *J* = 7.52 Hz, 1H, ArH), 8.01 (d, *J* = 8.16 Hz, 1H, ArH), 8.10 (s, 1H, NH), 8.37 (s, 1H, NH), 8.42 (d, *J* = 7.96 Hz, 1H, ArH), 8.45 (s, 1H, CH), 11.72 (s, 1H, NH): ^{13}C-NMR (100 MHz, DMSO-d_6): 124.43, 128.26, 128.36, 130.28, 133.27, 137.17, 148.22, 178.45; ^{15}N-NMR (40.55 MHz, DMSO-d_6) δ 8.10 (s, 1H, NH), 8.37 (s, 1H, NH), 11.72 (s, 1H, NH).

Entry 1: *5-(2,4,6-Trimethoxyphenyl)-1,2,4-triazolidine-3-thione*: ^{1}H-NMR (400 MHz, DMSO-d_6) δ 3.79 (s, 6H, $(OCH_3)_2$), 3.81 (s, 3H, OCH_3), 6.26 (s, 2H, ArH), 7.13(s, 1H, NH), 8.01 (s, 1H, NH), 8.26 (s, 1H, CH), 11.20 (s, 1H, NH): ^{13}C-NMR (100 MHz, DMSO-d_6): 55.40, 55.97, 91.06, 103.58, 138.44, 160.01,

162.22, 177.26; ^{15}N-NMR (40.55 MHz, DMSO-*d*6) δ 7.13 (s, 1H, NH), 8.01 (s, 1H, NH), 11.20 (s, 1H, NH); FT-IR: 1205, 1377, 1414, 1539, 1601, 2835, 2964, 3001, 3119, 3341, 3467.

Entry 2: *5-(2-Bromophenyl)-1,2,4-triazolidine-3-thione*: ^{1}H-NMR (400 MHz, DMSO-*d*6) δ 7.31 (t, *J* = 7.72 Hz, 1H, ArH), 7.39 (t, *J* = 7.36 Hz, 1H, ArH), 7.64 (d, *J* = 7.92 Hz, 1H, ArH), 8.08 (s, 1H, NH), 8.25 (t, *J* = 6.2 Hz, 1H, ArH), 8.27 (s, 1H, NH), 8.43 (s, 1H, CH), 11.62 (s, 1H, NH): ^{13}C-NMR (100 MHz, DMSO-*d*6): 123.47, 127.77, 127.80, 131.37, 132.94, 140.56, 178.23; ^{15}N-NMR (40.55 MHz, DMSO-*d*6) δ 8.06 (s, 1H, NH), 8.28 (s, 1H, NH), 11.54 (s, 1H, NH); FT-IR: 1274, 1370, 1462, 1509, 1606, 2979, 3019, 3150, 3241, 3412, 3746.

Entry 3: *5-(3,4-Dimethoxyphenyl)-1,2,4-triazolidine-3-thione*: 1H-NMR (400 MHz, DMSO-*d*6) δ 3.76 (s, 3H, OCH_3), 3.79 (s, 3H, OCH_3), 6.94 (d, *J* = 8.32 Hz, 1H, ArH), 7.12–7.15 (m, 1H, CH), 7.48 (s, 1H, NH), 7.96 (s, 1H, NH), 8.06 (s, 1H, NH), 11.25 (s, 1H, NH): ^{13}C-NMR (100 MHz, DMSO-*d*6): 55.45, 55.60, 108.35, 111.17, 122.21, 126.75, 142.75, 149.03, 150.54, 177.39; ^{15}N-NMR (40.55 MHz, DMSO-*d*6) δ 7.96 (s, 1H, NH), 8.06 (s, 1H, NH), 11.25 (s, 1H, NH); FT-IR: 1235, 1445, 1510, 1618, 2960, 3182, 3261, 3261, 3351.

Entry 4: *5-(4-Ethylphenyl)-1,2,4-triazolidine-3-thione*: 1H-NMR (400 MHz, DMSO-*d*6) δ 1.15 (t, *J* = 7.56 Hz, 3H, CH_3), 2.56–2.62 (m, 2H, CH_2), 7.22 (d, *J* = 8.16 Hz, 2H, ArH), 7.67 (d, *J* = 8.16 Hz, 2H, ArH), 7.91 (s, 1H, CH), 8.01 (s, 1H, ArH), 8.10(s, 1H, NH), 11.33 (s, 1H, NH): ^{13}C-NMR (100 MHz, DMSO-*d*6): 15.30, 28.03, 127.31, 128.08, 131.55, 142.60, 145.97, 177.71; ^{15}N-NMR (40.55 MHz, DMSO-*d*6) δ 7.91 (s, 1H, NH), 8.01 (s, 1H, NH), 11.33 (s, 1H, NH); FT-IR: 1289, 1368, 1457, 1533, 2927, 2955, 3024, 3153, 3247, 3403, 3650.

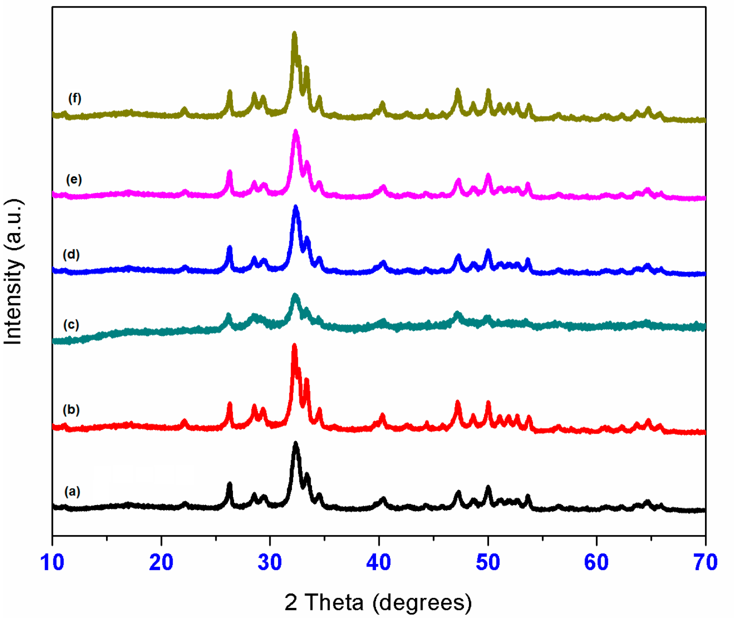

Figure S1. X-ray diffraction pattern of (**a**) FAp/without amino acid; (**b**) Sm-FAp/without amino acid; (**c**) Sm-FAp/Glutamic acid; (**d**) Sm-FAp/Aspartic acid; (**e**) Sm-FAp/Glycine; (**f**) Sm-FAp/Histidine before annealing at 350 °C.

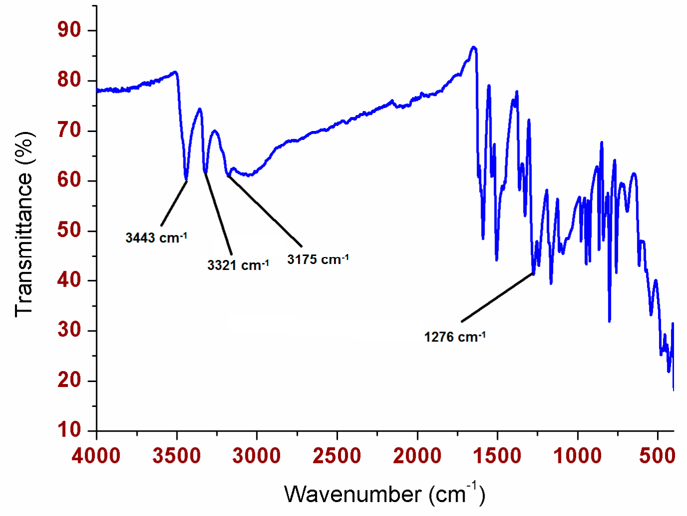

Figure S2. FT-IR specturum of 5-(2-nitrophenyl)-1,2,4-triazolidine-3-thione.

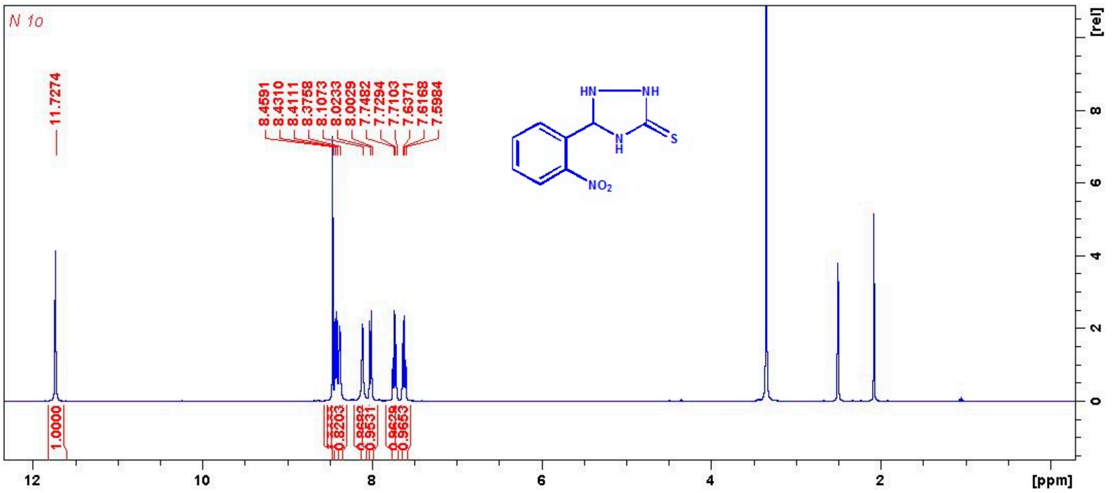

Figure S3. ^{1}H-NMR spectrum of 5-(2-nitrophenyl)-1,2,4-triazolidine-3-thione.

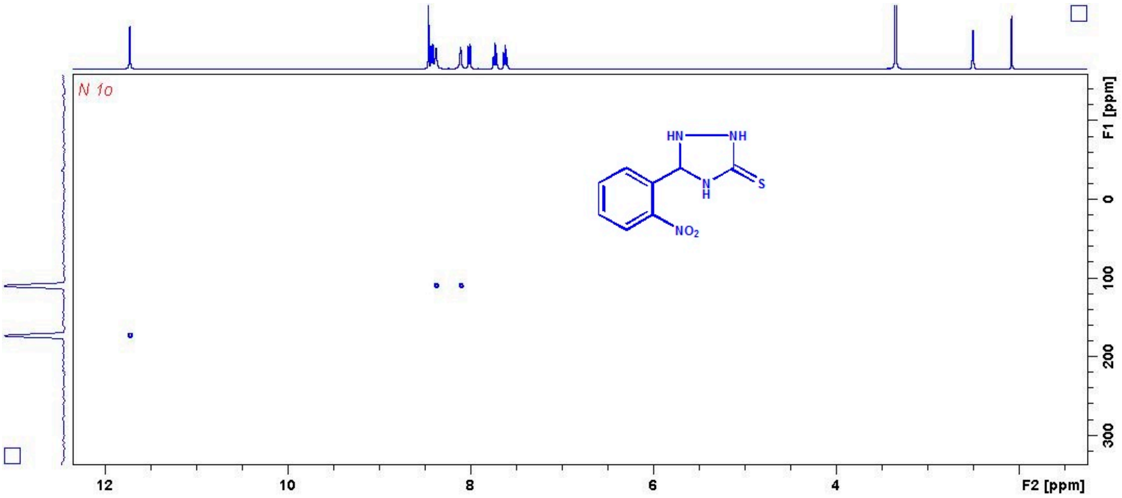

Figure S4. ^{15}N-NMR spectrum of 5-(2-nitrophenyl)-1,2,4-triazolidine-3-thione.

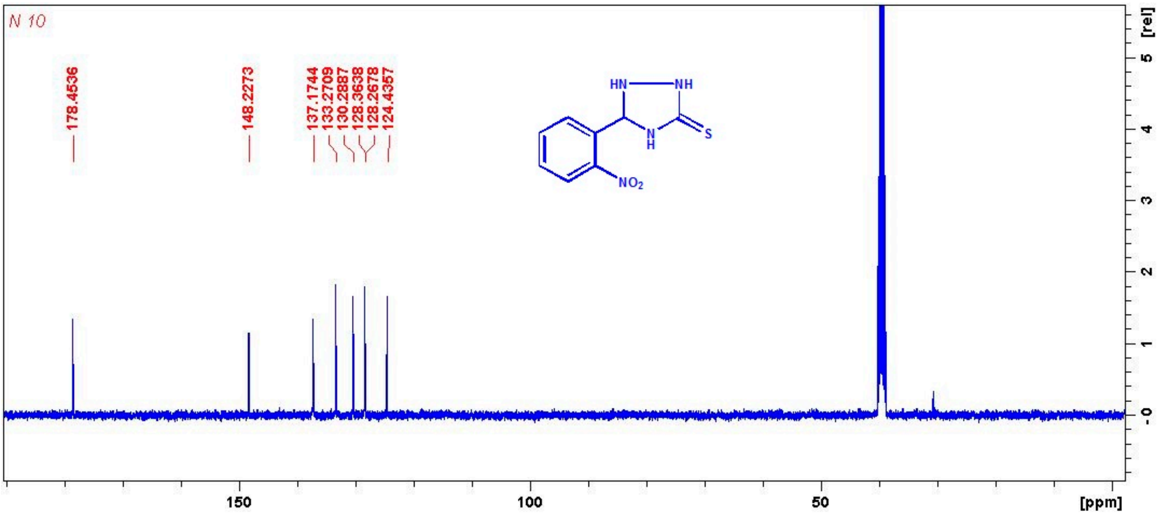

Figure S5. ^{13}C-NMR spectrum of 5-(2-nitrophenyl)-1,2,4-triazolidine-3-thione.

Entry 1: ^{1}H-, ^{13}C-, ^{15}N-NMR Spectra

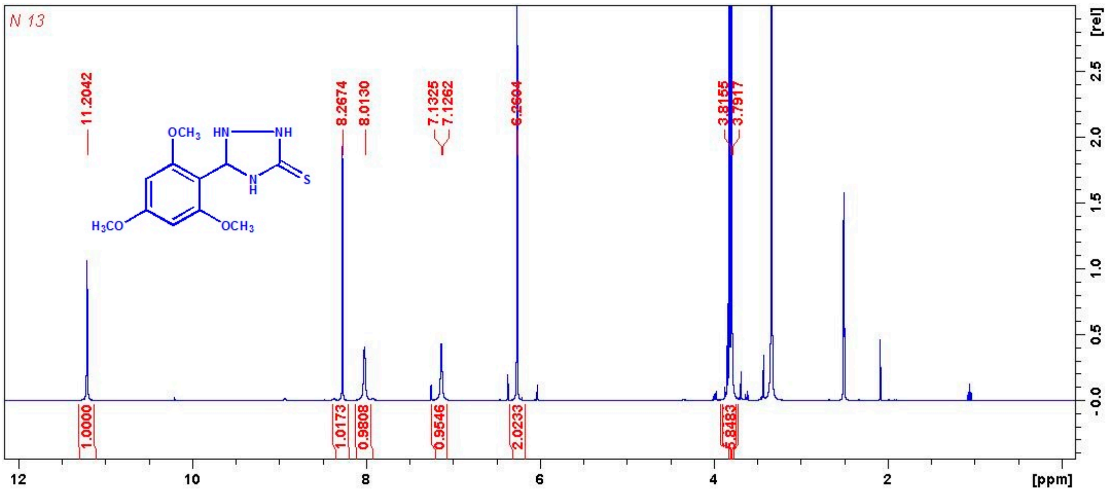

Figure S6. ^{1}H-NMR spectrum of 5-(2,4,6-trimethoxyphenyl)-1,2,4-triazolidine-3-thione.

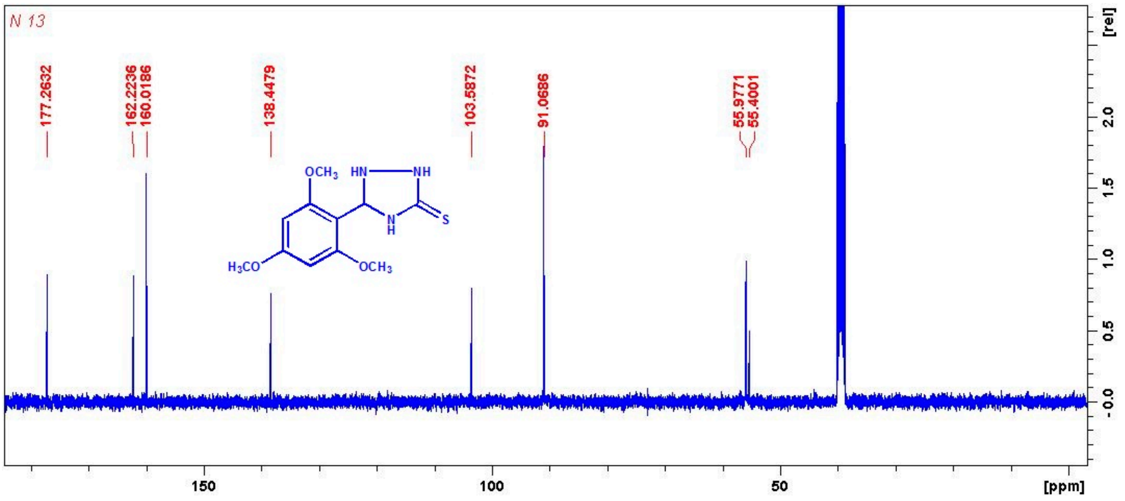

Figure S7. ^{13}C-NMR spectrum of 5-(2,4,6-trimethoxyphenyl)-1,2,4-triazolidine-3-thione.

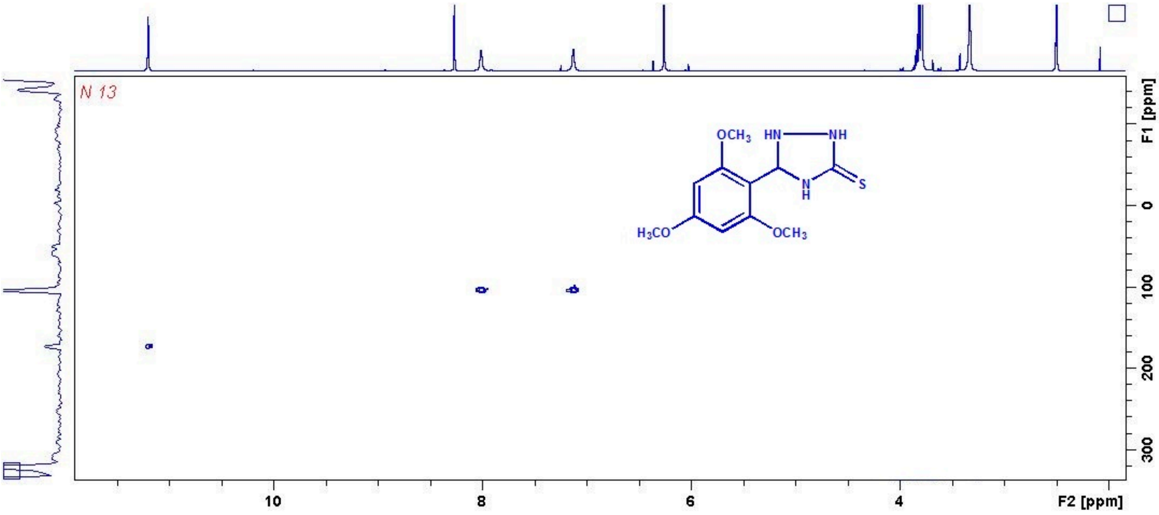

Figure S8. ^{15}N-NMR spectrum of 5-(2,4,6-trimethoxyphenyl)-1,2,4-triazolidine-3-thione.

Entry 2: ^{1}H-, ^{13}C-, ^{15}N-NMR Spectra

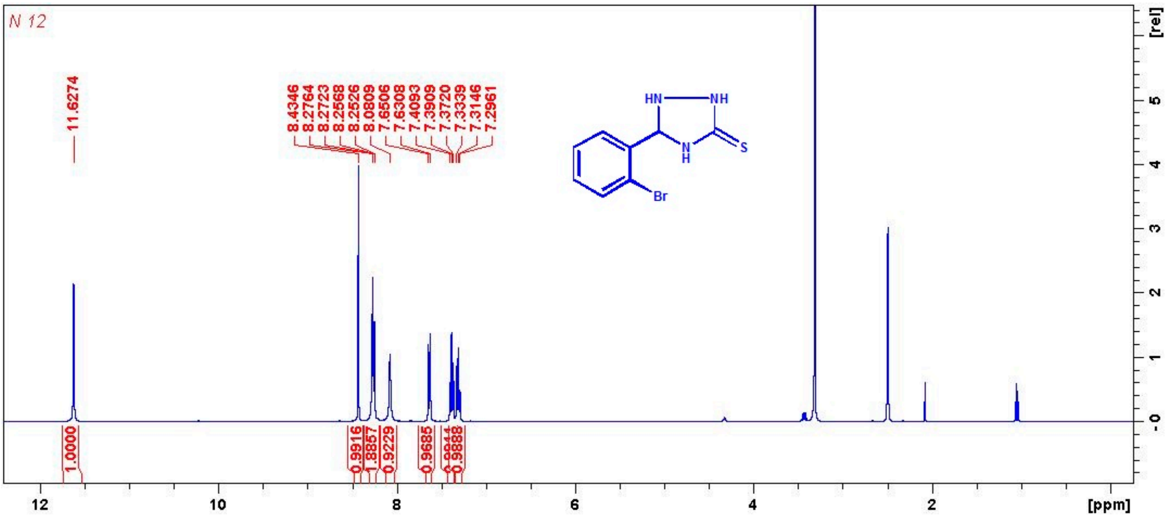

Figure S9. ^{1}H-NMR spectrum of 5-(2-bromophenyl)-1,2,4-triazolidine-3-thione.

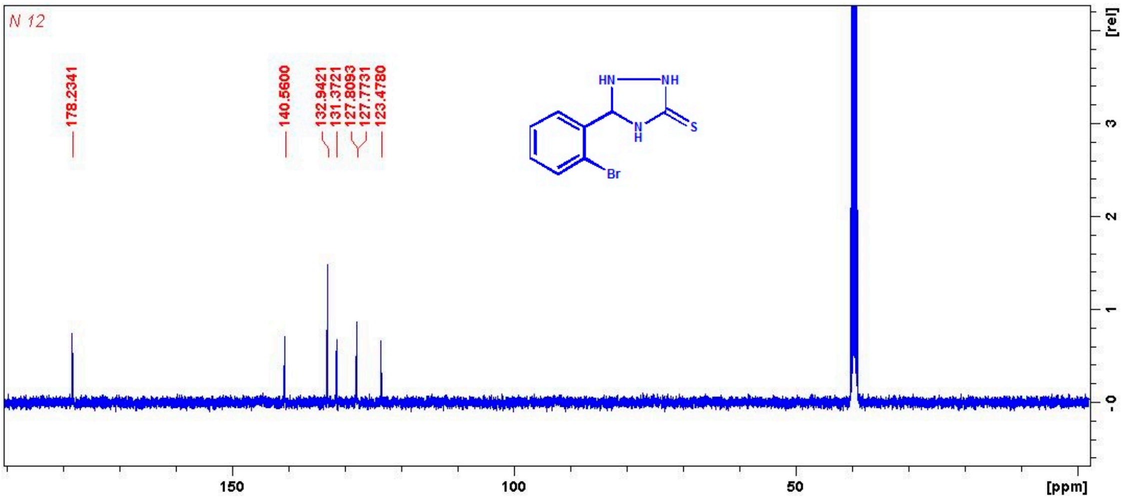

Figure S10. ^{13}C-NMR spectrum of 5-(2-bromophenyl)-1,2,4-triazolidine-3-thione.

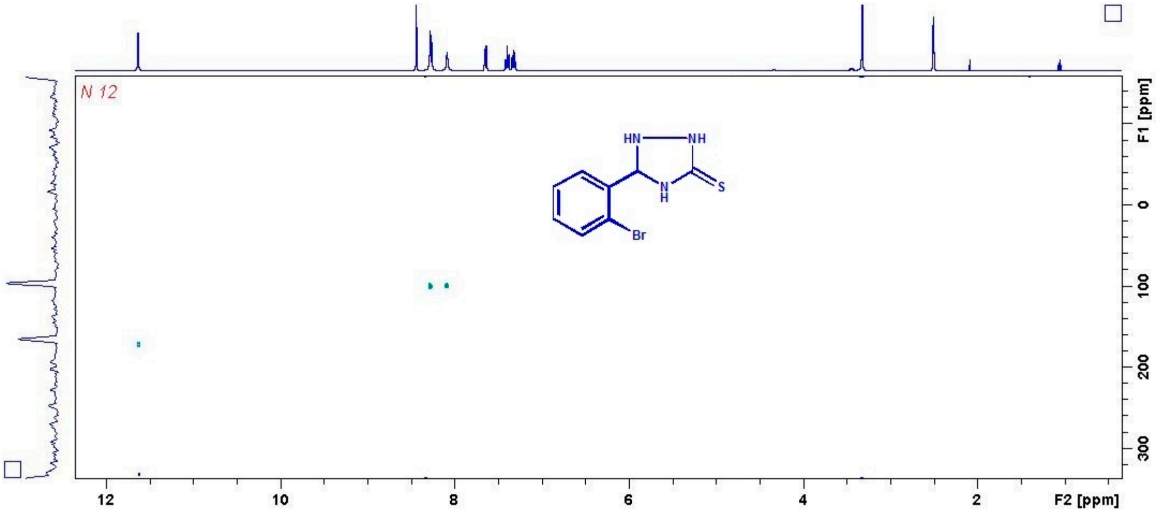

Figure S11. ^{15}N-NMR spectrum of 5-(2-bromophenyl)-1,2,4-triazolidine-3-thione.

Entry 3: ^{1}H-, ^{13}C-, ^{15}N-NMR Spectra

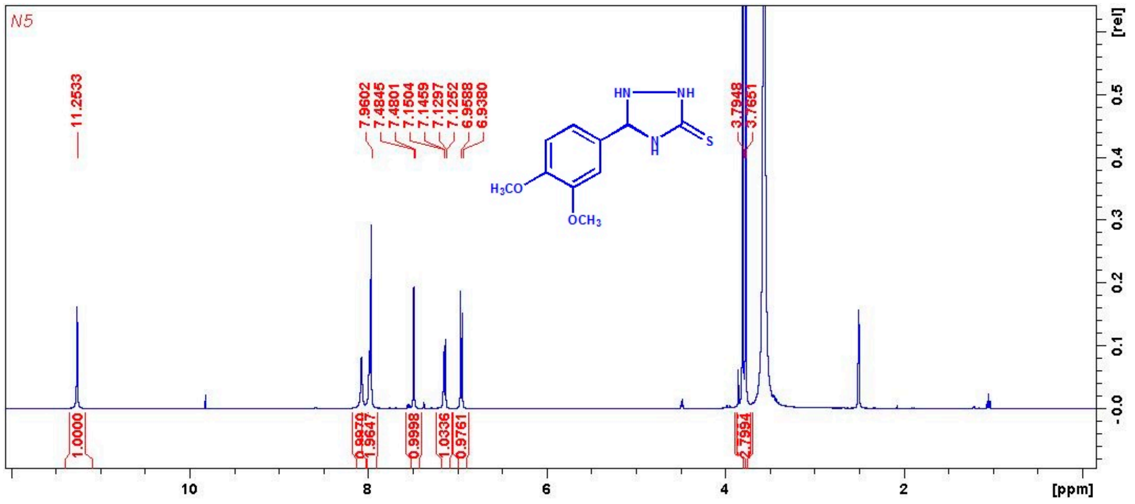

Figure S12. ^{1}H-NMR spectrum of 5-(3,4-dimethoxyphenyl)-1,2,4-triazolidine-3-thione.

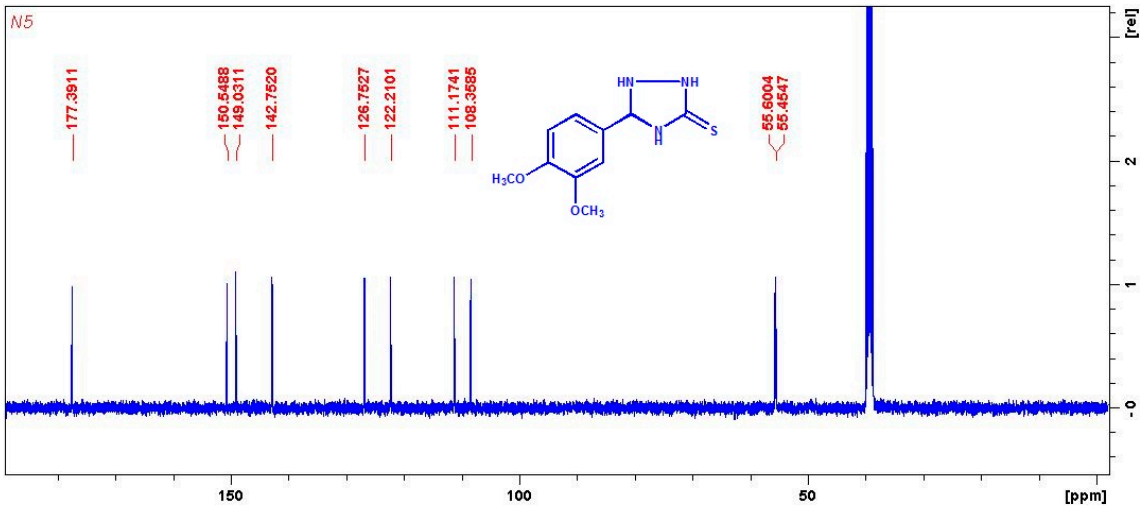

Figure 13. ^{13}C-NMR spectrum of 5-(3,4-dimethoxyphenyl)-1,2,4-triazolidine-3-thione.

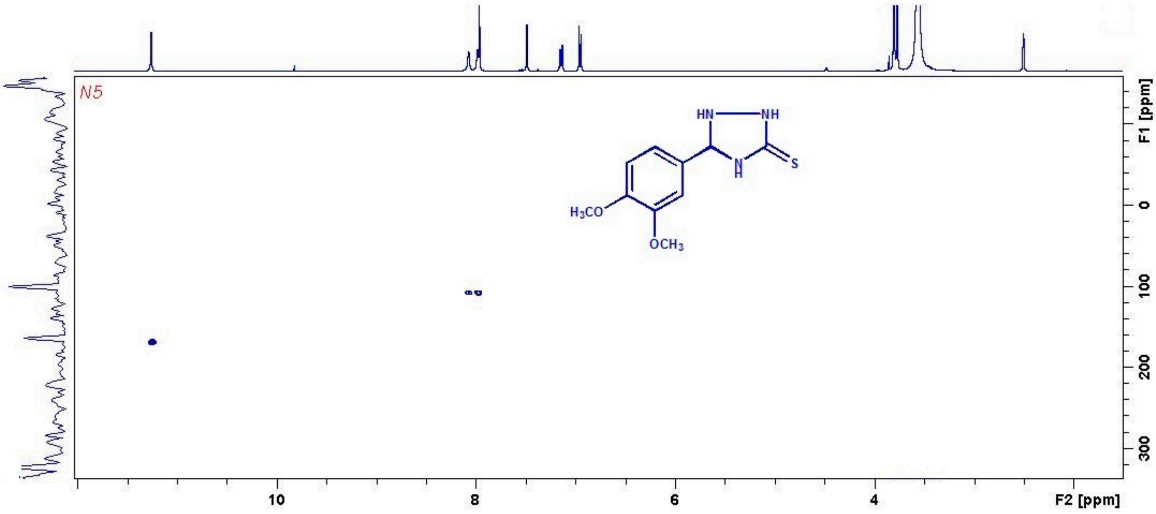

Figure 14. ^{15}N-NMR spectrum of 5-(3,4-dimethoxyphenyl)-1,2,4-triazolidine-3-thione.

Entry 4: ^{1}H-, ^{13}C-, ^{15}N-NMR Spectra

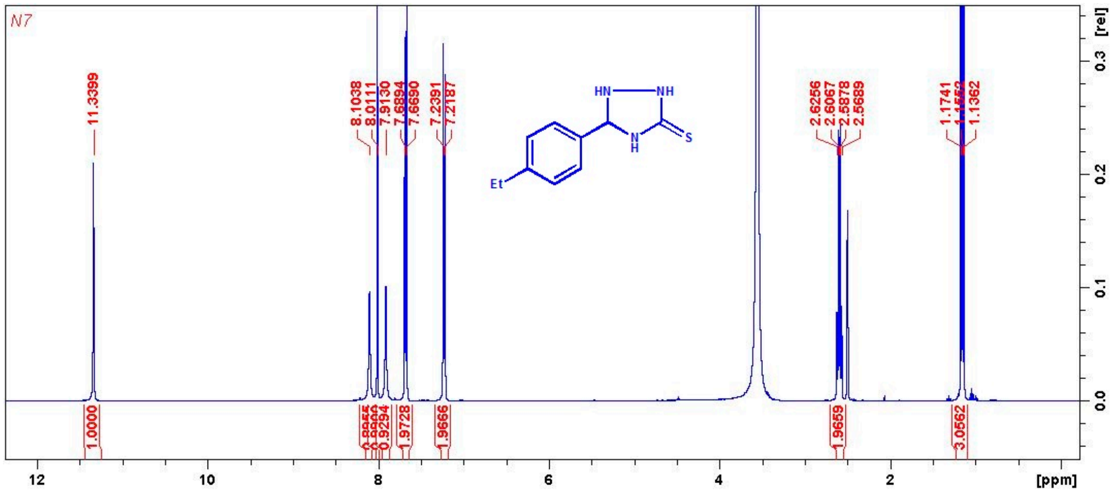

Figure 15. ^{1}H-NMR spectrum of 5-(4-ethylphenyl)-1,2,4-triazolidine-3-thione.

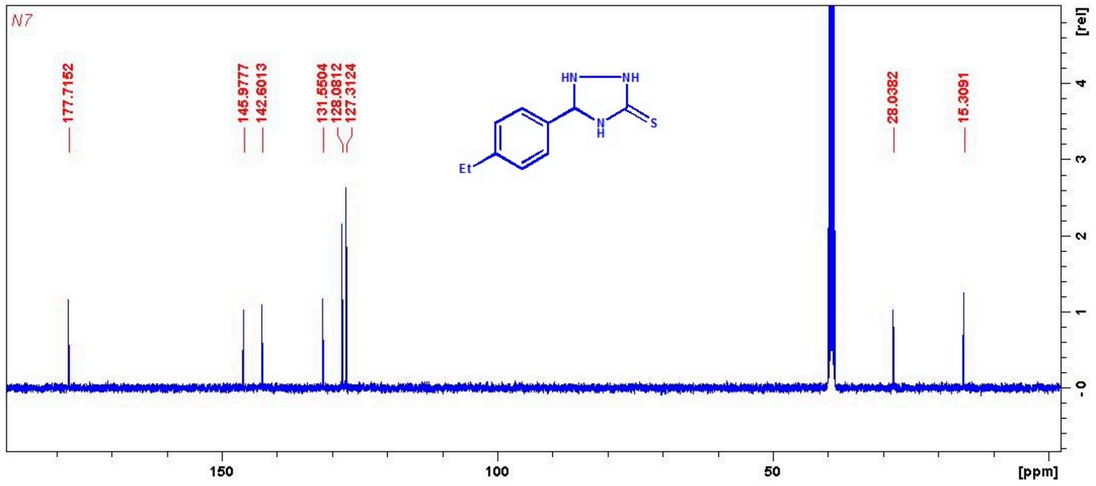

Figure 16. ^{13}C-NMR spectrum of 5-(4-ethylphenyl)-1,2,4-triazolidine-3-thione.

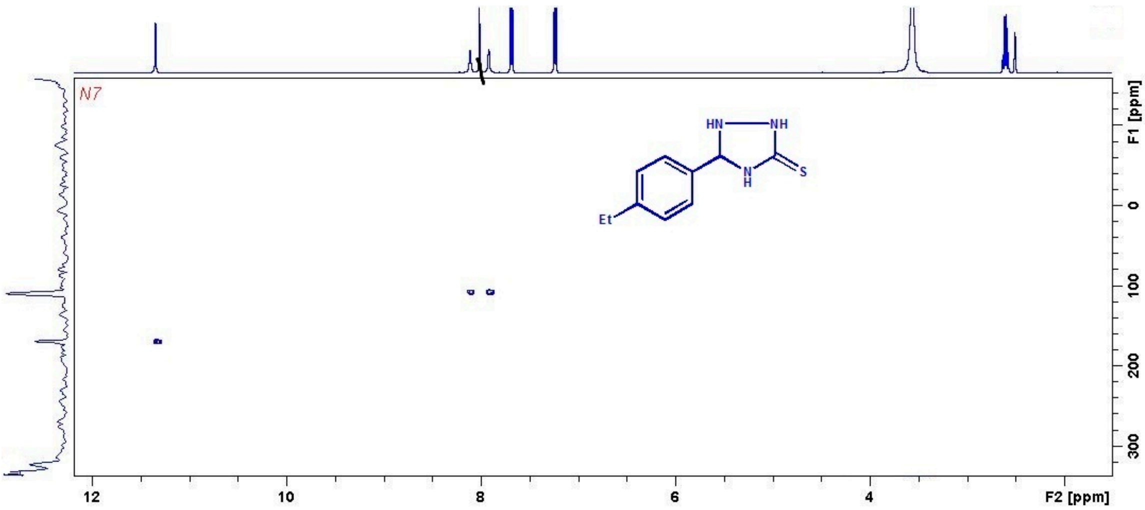

Figure S17. ^{15}N-NMR spectrum of 5-(4-ethylphenyl)-1,2,4-triazolidine-3-thione.
